# Gene expression-based biomarkers for discriminating early and late stage of clear cell renal cancer

**DOI:** 10.1038/srep44997

**Published:** 2017-03-28

**Authors:** Sherry Bhalla, Kumardeep Chaudhary, Ritesh Kumar, Manika Sehgal, Harpreet Kaur, Suresh Sharma, Gajendra P. S. Raghava

**Affiliations:** 1 Bioinformatics Centre, CSIR-Institute of Microbial Technology, Sector 39A, Chandigarh-160036, India; 2 CSIR-Central Scientific Instruments Organization, Sector 30C, Chandigarh-160030, India; 3 Centre for Systems Biology and Bioinformatics, Panjab University, Sector 14, Chandigarh-160014, India

## Abstract

In this study, an attempt has been made to identify expression-based gene biomarkers that can discriminate early and late stage of clear cell renal cell carcinoma (ccRCC) patients. We have analyzed the gene expression of 523 samples to identify genes that are differentially expressed in the early and late stage of ccRCC. First, a threshold-based method has been developed, which attained a maximum accuracy of 71.12% with ROC 0.67 using single gene *NR3C2*. To improve the performance of threshold-based method, we combined two or more genes and achieved maximum accuracy of 70.19% with ROC of 0.74 using eight genes on the validation dataset. These eight genes include four underexpressed (*NR3C2, ENAM, DNASE1L3, FRMPD2*) and four overexpressed (*PLEKHA9, MAP6D1, SMPD4, C11orf73*) genes in the late stage of ccRCC. Second, models were developed using state-of-art techniques and achieved maximum accuracy of 72.64% and 0.81 ROC using 64 genes on validation dataset. Similar accuracy was obtained on 38 genes selected from subset of genes, involved in cancer hallmark biological processes. Our analysis further implied a need to develop gender-specific models for stage classification. A web server, CancerCSP, has been developed to predict stage of ccRCC using gene expression data derived from RNAseq experiments.

Renal cell carcinoma (RCC) is the most prevalent kidney cancer accounting to high mortality rates globally. According to the US cancer statistics, there is an estimate of 63,990 new cases and 14,400 deaths in 2017, which makes it a life-threatening disease^1^. This cancer results from uncontrolled growth of cells lining the proximal convoluted tubule, involved mainly in the transport of waste molecules to the urine. Primarily, renal cancer is categorized in four subcategories based on its appearance under a microscope, which includes chromophobe, clear cell, collecting duct and papillary. The clear cell renal cell carcinoma (ccRCC) is the major contributor to renal cell carcinoma (80%) among the different forms of kidney cancers. Fatality rates are greater when the cancer is discovered in the late stages whereas early detection coupled with effective treatment has been linked to higher survival rates^2^. Thus, development of an economic and efficient strategy to identify the stage of cancer is important to understand the severity of a patient^3^. Currently, major techniques for screening and staging of cancer includes imaging techniques (CT, MRI and bone scan)^4^ and TNM staging system^5^. These techniques have their limitations; thus there is a need to develop alternate methods for classification.

Fortunately, recent advancements in high-throughput DNA sequencing technology have made whole cancer genome sequencing conceivable in substantial time and at a reasonable expense. It has facilitated simple and effective recognition of commonly mutated, amplified, inserted and deleted genes across diverse cancer types^6^. Recently, classification models have been developed to distinguish early stage and late stage of ccRCC using the expression of 62 genes^7^. Authors developed different models in the study and evaluated them based on 10-fold cross-validation and independent validation dataset. They achieved maximum accuracy of 76.84% and ROC of 0.77 on the independent dataset.

The current study endeavors to evaluate minimum number of potential markers, which can define the stage progression from early to late stage of cancer using gene expression data derived from experiments in the form of RNA-Seq by Expectation Maximization (RSEM) values. In order to assess the robustness of biomarkers, we also carried out resampling of the entire dataset for 100 times for selecting stable features. We aimed to complement previous *in silico* techniques for achieving better performance and rank individual genes based on their discriminating power. Once, the potential biomarker genes were identified, we extracted comprehensive information about these genes from literature to understand their role in various biological processes. This study also highlights the importance of gender-specific models for discriminating early and late stage samples. Ultimately, we have developed a web resource, CancerCSP based on prioritization of essential genes; depending upon the gene expression that could successfully differentiate early/late stage of ccRCC.

## Results

### Expression-threshold based features

#### Single gene biomarkers

In order to prioritize genes, which express differently in the early and late stage of cancer, we developed threshold-based models using each gene. These models are called single gene-based threshold models as they use the expression of a single gene at a time. We computed the performance of all 19,166 genes to rank these genes based on their performance. This way, we were able to rank the genes based on their performance to classify early and late stage of cancer. Out of the 19,166 genes, analysis of 20 genes including 10 overexpressed and 10 under-expressed genes and their involvement in cancer hallmark biological processes is shown in Table 1 wherein Nuclear Receptor Subfamily 3 Group C Member 2 (*NR3C2*) gene shows the highest performance in classification (ROC 0.67 with an accuracy of 71.12%) for training data. *NR3C2* is overexpressed in early stage of ccRCC. This analysis propose that when the normalized RSEM score of *NR3C2* is greater than the threshold of −0.48, then there are chances that cancer is in early stage, and if it is less than −0.48, the cancer is in late stage. This type of analysis clearly exhibits the contribution of each gene as a putative marker to predict early stage of ccRCC.

#### Multiple genes biomarkers

Earlier, we described single gene-based threshold models, while this section focuses on multiple-gene based threshold models. In these models, expression of two or more genes is used as an input feature. Based on single gene threshold-based methods, we identified top 50 genes from 19,166 genes with the highest ROC. The correlation matrix for 50 genes was calculated and if any combination of gene had correlation greater than 0.6, then the gene with lower ROC was removed. After removing correlated genes, we obtained 28 out of 50 genes and named the set as RCSP-set-Threshold. The expressions of these 28 genes were used as input feature to develop machine-learning models to discriminate early and late stage of cancer. As shown in Table 2, SVM based model achieved maximum performance with ROC 0.78 and accuracy 73.27% on training dataset when evaluated using ten-fold cross-validation. We also evaluated performance of the above model on independent or external validation dataset and achieved maximum ROC of 0.77 with accuracy of 71.15%.

**Figure 1:**
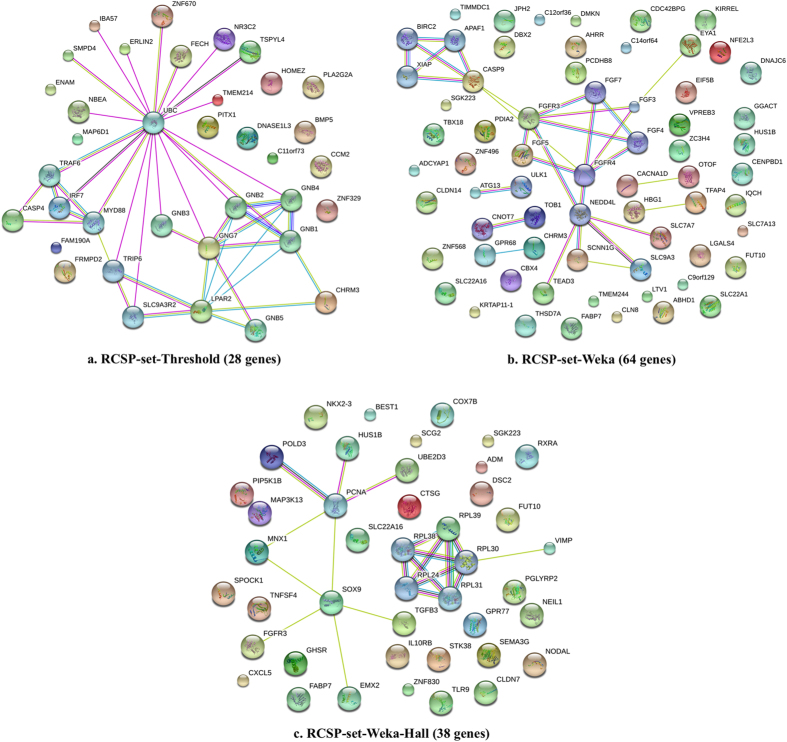
The protein–protein interaction network among the potential ccRCC biomarkers generated using STRING database (with direct and indirect interactions) ((**a**) for RCSP-set-Threshold, (**b**) for RCSP-set-Weka, and (**c**) for RCSP-set-Weka-Hall).

In order to understand the significance of these selected genes in the biological processes, we performed interaction analysis of these 28 proteins. As shown in Fig. 1A, three proteins encoded by *GNG7, LPAR2* and *CHRM3* genes depicted direct interactions. These genes are major components of the phosphoinositide 3-kinase (PI3K)-Akt signaling pathway, which is known to be mutated in ccRCC patients as per the TCGA analysis^8^. After including the indirect interactions (no more than 10 interactors in first shell) among the 28-gene dataset, the interaction network revealed a hub node ubiquitin (*UBC*), pointing to the major role of ubiquitination in renal cancer. Usually, *UBC* is implicated in protein degradation, cell cycle regulation, DNA repair and is identified to contribute towards cancer metastasis^9^. The pathway analysis for renal carcinoma differentiating normal and cancer markers have also mentioned *UBC* as a vital player regulating numerous proteins^10^. In addition, a significant network pattern comprising of *GNB1, GNB2, GNB3, GNB4, GNB5* and *GNG7* proteins was spotted. All these proteins are members of G protein family and govern major signaling cascades by transmitting signals from receptors to the effector proteins.

In the next analysis, we separated the above 28 genes into two groups; (i) Group-A containing 16 genes which are overexpressed in the early stage, and (ii) Group-B containing 12 genes, which are overexpressed in the late stage of cancer. Next, we developed threshold-based models using more than two genes and identified the best set of genes from group A and B. For this purpose, we performed analysis on the genes of Group A, where the expression of top ranked gene is combined with the remaining 15 genes in an iterative manner and subsequently identified the best pair of genes. As described in Methods, the threshold-based model utilizes mean expression of genes to classify the stage of ccRCC samples. This best pair of genes is then combined with other genes one-by-one to identify the best three genes and so on. Finally, we obtained the best four genes i.e. *NR3C2, ENAM, DNASE1L3* and *FRMPD2* (setA-1) from group A genes. The same exercise was also repeated for the genes of group B providing the four best genes i.e. *PLEKHA9, MAP6D1, SMPD4* and *C11orf73* (setB-1) (Supplementary Table S1). We selected only up to four genes as the performance was not increasing further with the increase in number of genes. Moreover, we developed different types of prediction models using setA-1 and achieved ROC 0.76 for SVM models on the training dataset. We also evaluated the performance on an independent dataset and achieved similar performance ROC 0.80. Similarly, for setB-1, we attained maximum ROC 0.74 on the training dataset and validation data. On combining setA-1 and setB-1 (i.e., Combo-1), ROC 0.77 and 0.80 was obtained on training dataset and external validation dataset respectively (Table 3).

### Machine Learning based Features

In this section, we explain the selected features based on the performance of models developed using machine-learning techniques. Instead of combining two genes by taking simple average (as done in previous section of multiple genes biomarkers), here we combined the two genes using SVM. In the previous section, we combined the expression of genes for developing the threshold-based method for more than one gene. Here, we developed SVM based models using best two genes, three genes, four genes and so on, to further identify the minimum number of features for obtaining the best model. First, genes of Group-A (16 genes) were used for developing the two genes based SVM model, and we then discovered the best pair of genes for developing SVM model. Similarly, we searched the third gene with the best pair of genes, which gave best SVM model. This process was repeated till the performance was saturated, and we obtained the best SVM model using five genes (setA-2). Similar procedure was repeated for Group-B genes to find the five best genes (setB-2) (Supplementary Table S2). The SVM model using best setA-2 achieved maximum ROC of 0.75 with an accuracy of 70.88% on training dataset and accuracy of 67.31% and ROC of 0.75 on validation dataset (Table 4). As shown in Table 4, SVM model based on setB-2 gave a maximum accuracy of 69.69% and ROC of 0.76 on training dataset and accuracy of 64.42% and ROC of 0.72 on the validation dataset. In order to increase the performance, we further combined the best genes from Group A and B and pooled the best ten genes (Combo-2). The SVM model based on these ten genes got a maximum accuracy of 72.62 and ROC of 0.78 on training data and accuracy of 70.19 with ROC of 0.77 on the validation dataset (Table 4).

**Figure 2:**
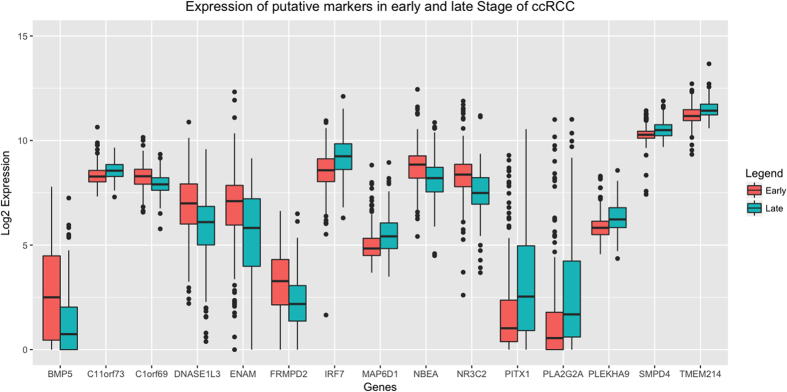
A box plot diagram representing median log expression distribution of 15 genes differentially expressed in early and late stage of ccRCC with a p-value < 0.01 calculated using Wilcoxon rank-sum test. These genes are the union of Combo-1 and Combo-2 sets.

In total, the threshold-based and SVM-based features provided 15 genes. Their normalized log2 RSEM expression distribution in the early and late stage is shown in Fig. 2 in the form of boxplots. The *p*-value is calculated using Wilcoxon rank-sum test. The computed *p*-value is less than 0.01 in all the cases that show a significant difference in normalized expression values of these genes in early and the late stage ccRCC.

### Weka Based Features

In this section, the feature selection was performed by Weka and the number of features was reduced from 19,166 to 64 features (RCSP-set-Weka). We used features from RCSP-set-Weka (Supplementary Figure S1) for developing models based on different machine-learning algorithms where SVM-based model achieved maximum ROC of 0.83 with accuracy 78.18% on training dataset (Table 5) and ROC of 0.81 with accuracy 72.64% on the validation dataset (Table 5).

We performed the interaction analysis of RCSP-set-Weka using STRING database and found the significance of various markers such as *CASP9, FGFR3, FGF5, CHRM3* and *GPR68* depicting direct interactions and *ATG3, ULK1, CNOT7, TOB1, HBG1* and *TFAP4* portraying indirect interactions (no more than 10 interactors in first shell) through various levels of regulations (Fig. 1B). *ULK1* and *ATG3* are autophagy associated genes wherein the components of the process are well deciphered in chronic kidney disease by affecting the mTOR pathway^11^. A conserved pattern comprising of *FGF3, FGF4, FGF5, FGF7* and *FGFR4* proteins belonging to a family of fibroblast growth factors is also found in the network. These proteins are key players in cell proliferation, differentiation, and signaling pathways and have critical involvement in developmental processes. In addition, these proteins are also considered as potential targets for designing therapies against RCC^12^,^13^.

### Cancer Hallmark GO-term based models

In order to encompass a comprehensive list of cancer markers, we also selected a subset of genes, which are components of cancer hallmark processes like apoptosis, DNA repair, cell cycle, cell adhesion, cell growth, phosphorylation, response to external stimulus, cell motility and immune response. We got 38 (RCSP-set-Weka-Hall) features from 4,843 genes using Weka and achieved an accuracy of 77.7% with ROC 0.83 on training data and accuracy of 72.64% with ROC of 0.78 on the validation data (Table 6). The accuracy obtained on validation dataset from the cancer RCSP-set-Weka-Hall was the same as obtained from the RCSP-set-Weka. Hence, this feature selection is more reliable as the search space has been refined to lesser number of features. This method used only those genes (Supplementary Table S3) which are covered in cancer hallmark GO processes.

Figure 1C evidently represents the interactions (direct and no more than 5 interactors in first shell) from the cancer hallmark GO term related proteins. A number of key players in ccRCC are spotted in the network with *PCNA* and *SOX9* as hub proteins controlling the operations of many neighboring proteins like *POLD3, HUS1B* and *MNX1. PCNA* is a well known molecular marker for proliferation^14^ and SOX9 is known to be upregulated in metastatic renal cell carcinoma and causes drug resistance in cancer by activating Raf/MEK/ERK pathway^15^. Most of the proteins are well established markers for renal cancer progression^16^ as they are directly extracted from cancer hallmark processes. A conserved sub-network of ribosomal proteins is also observed in the interaction map demonstrating the elevated need of proteins required for tumor proliferation.

We also built models combining clinical features (Age and Gender) with RCSP-set-Weka and RCSP-set-Weka-Hall selected features but no substantial enhancement in the performance was observed (Supplementary Figures S2 and S3). In addition, we combined the three types of features *i.e*. RCSP-set-Threshold features, RCSP-set-Weka features and RCSP-set-Weka-Hall selected features into various combinations but there was just a marginal rise in the performance (Supplementary Table S4).

### Gender-based Classification Models

It has been widely studied that there are gender-specific differences in the development and survival of various tumors^17^,^18^. We analyzed if there is a requirement of different stage-specific putative markers for ccRCC in the case of males and females. We computed average expression of each gene in 200 early and 138 late stage male samples. Supplementary Table S5 depicts the top 10 genes that have a maximum difference in average expression in the early and late stage of male ccRCC samples. Similarly, top ten genes having a maximum difference between early and late stage of female samples is shown in Supplementary Table S6. As shown in Supplementary Tables S5 and S6, only two genes were shared between top 10 genes in male and female samples. The difference in these genes indicates that genomic expression is regulated differentially in males and females.

Thus, we developed gender-specific models for classification of ccRCC. First, the male-specific models were developed using 80% male samples (159 early and 109 late stage). The Weka-based feature selection method (as discussed in Methods) was used for selecting 64 features. These features were used for developing models and got maximum ROC 0.87 with an accuracy of 80.22% (Table 7) on training dataset and 77.14% accuracy with ROC of 0.80 on validation dataset (41 early stage and 29 late stage samples) using SVM. Second, classification models were trained on female samples (93 early and 54 late stage) where ROC 0.90 with accuracy 85.71% was achieved (Table 7) using SVM. 78.95% accuracy and 0.82 ROC was obtained on validation dataset of females (24 early stage samples and 14 late stage samples). These results indicate that gender-specific models can be more specific. However, to make this conclusion concrete, we require a significant number of samples separately for male and female categories. Only one gene, *CTSG* is common between male and female specific biomarkers that points to the different regulation of tumor microenvironment in different genders.

The male and female protein-protein interaction network (Supplementary Figure S4) reveals the significance of markers involved in different cellular processes. In the female dataset, *GAPDH* is acting as a hub node connecting many proteins like *CTSG, STAT2, GCLC* and *WWOX*, thus regulating numerous proteins and is considered a critical component of apoptosis^19^. The proteins in this network are primarily regulating multiple signaling cascades such as MAP kinase, RAS signaling, and PI3K/AKT signaling. When expanding the network connectivity (no more than 5 interactors in first shell), it depicts association with a complex network of proteins from the ribosomal protein family (*RPL24, RPL30, RPL31, RPL38* and *RPL39*). These ribosomal proteins play a vital role in cancer progression by directing elevated protein synthesis in cancer proliferation^20^,^21^. Whereas the male dataset provides lesser direct interactions and constitutes mainly of indirect interactions (no more than 5 interactors in first shell) involving a family of proteins *COPB1, COPB2, COPA* and *COPG1* which are involved in intracellular transport. These biomarkers are also vital components of cell signaling and cell proliferation processes.

### Functional enrichment of deduced markers

**Figure 3:**
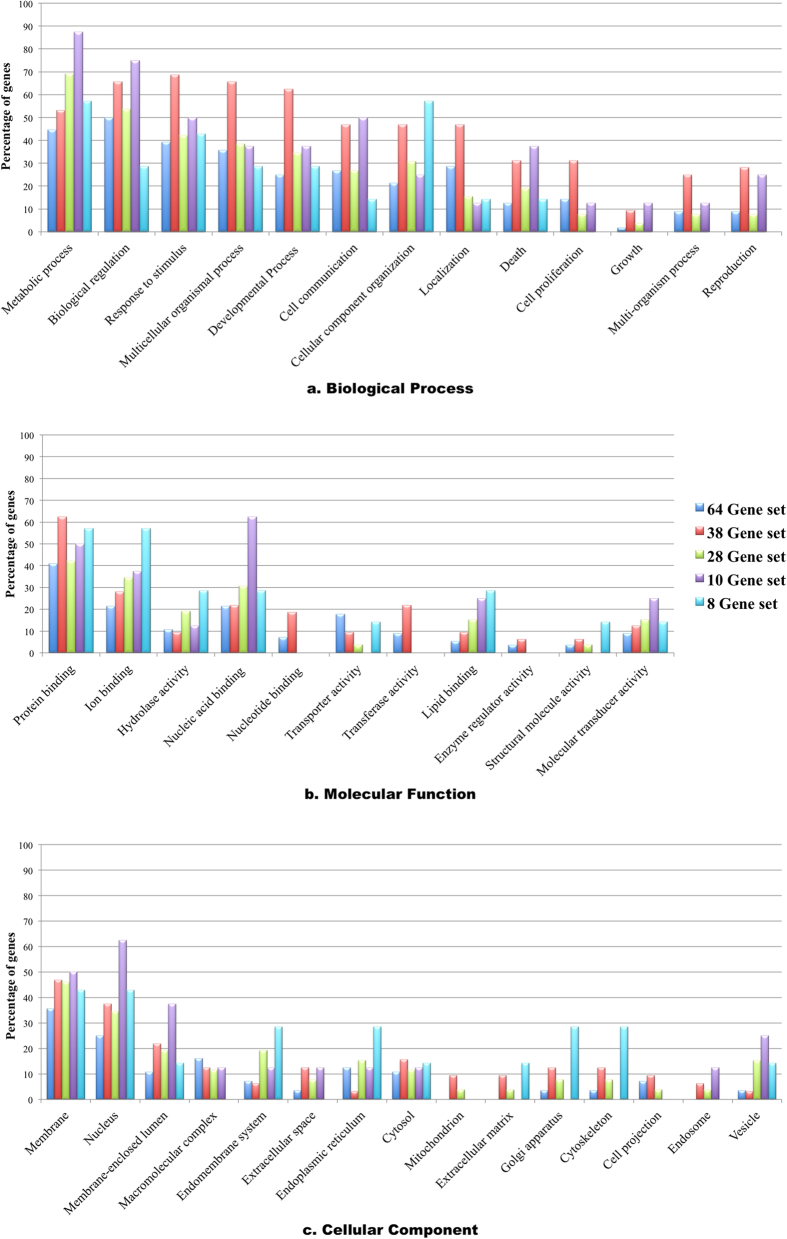
The gene ontology analysis depicting percentage distribution of different biomarkers in major biological processes, molecular functions and cellular components from the five gene sets. In the process of gene enrichment, 56 out of 64 genes, 32 out of 38 genes, 26 out of 28 genes, 8 out of 10 genes and 7 out of 8 genes were annotated respectively.

A comprehensive search was perceived for all the categories comprising of 64 (RCSP-set-Weka), 38 (RCSP-set-Weka-Hall), 28 (RCSP-set-Threshold), 8 (Combo-1), 10 (Combo-2). Figure 3 represents the comparative gene ontology information on all the characterized markers where a majority of genes were implicated in metabolic processes, biological regulation, protein binding, ion binding and were localized mainly in the membrane and nucleus. The inferred potential markers are involved in essential cellular processes and signaling pathways like VEGFR, PDGFR-beta, PAR1-mediated thrombin, IL5-mediated and mTOR signaling; thus, governing vital cancer-related processes such as cell growth, proliferation, motility, and survival. The dataset subjected to enrichment analysis also revealed the involvement of *CTSG* and *NR3C2* in ACE inhibitor pathway, which is already extensively studied for RCC.

In addition, the exploration of regulatory elements such as transcription factors (TFs) assisted in comprehending the regulatory pattern observed by these markers; revealing multiple binding associations of a few TFs with key candidates reported in the study. *STAT5A, HFH3, NFAT, FOXO4*, and *IRF1* are some of the TFs that bind to multiple targets derived from the acquired ccRCC markers. *IRF1* alone is believed to regulate the expression of *NR3C2, PITX1, DNASE1L3* and *BMP5* genes by binding to interferon stimulated regulatory element (ISRE) in the promoter of these genes.

The gene enrichment analysis was also performed for gender-specific differentiation of markers where the majority of proteins in males were involved in developmental processes, cell communication, multicellular organismal processes and proliferation processes as compared to females. Subsequently, these results portray increased susceptibility of males for developing ccRCC which is in harmony with the global statistics for ccRCC. A parallel analyses on its cellular component shows that male related proteins are mainly localized in nucleus, endomembrane system and envelopes whereas female related proteins are mainly components of a macromolecular complex (Supplementary Figure S5). The process of cell proliferation dominates in males while female dataset marginally highlighted the significance of cell death process.

### Web Server Implementation

In order to assist the scientific community, we developed a web server, CancerCSP (clear cell renal cancer stage prediction), that implements models developed in the current study for analysis and prediction of cancer stage from the gene expression data. This server has two major modules; one for predicting the cancer stage, and another for the analysis of gene expression data.

#### Prediction Module

This module allows the users to predict the cancer stage of their sample using RSEM gene expression quantification values. The user needs to provide gene expression (RSEM values) of biomarker genes for every patient. The number of patients corresponds to the number of columns in a file. The output includes a list for patient and corresponding predicting stage of cancer (early or late stage). The user can choose among the models developed from RCSP-set-Weka, RCSP-set-Weka-Hall, Combo-1 and Combo-2 sets, which have been deduced as putative biomarkers sets for stage progression of ccRCC.

#### Data Analysis Module

The gene statistics module is helpful in evaluating the role of each gene in discrimination of early stage from the late stage. This module gives *p*-value (calculated using Wilcoxon rank test) for each gene that signifies whether the gene expression in early and late stage varies significantly. It also gives threshold-based ROC of each gene along with average expression of that gene in the early and late stage of cancer. This module also provides normalized threshold score of each gene and converts the RSEM value to a *z*-score so that user can compare whether the *z*-score is above or below the threshold. This web server is available from URL http://crdd.osdd.net/raghava/cancercsp/ for public use.

## Discussion

The main aim of this study was to find a signature panel with a minimum number of genes that can reasonably discriminate early and late stage of ccRCC patients using gene expression data. We ranked each gene based on performance (ROC value) of their classification models to distinguish the early and late stage of cancer. Interestingly, *NR3C2* gene has shown highest classification ROC of 0.67 using threshold-based model individually. *NR3C2* has previously been described as tumor suppressor gene, and its role has already been demonstrated in renal and pancreatic cancer^22^,^23^. Reduced level of this gene has shown poor survival in pancreatic cancer patients^24^. It has also been found to be downregulated in five types of cancer in TCGA^25^. Further, a list of top 28 genes (RCSP-set-Threshold) is proposed which classify early and late patients with ROC of 0.77. The genes in this panel have been implicated in the PI3K-Akt signaling pathway as per the enrichment analysis, which is analogous to the TGCA mutation analysis for ccRCC. The network analysis using STRING database pointed out that the genes in our RCSP-set-Threshold panel indirectly interact with *UBC* stating concordance with a previous network study on renal cancer^10^. We further extracted RCSP-Combo-1 (8 genes) and Combo-2 (10-genes) from RCSP-set-Threshold. According to our analysis, *DNASE1L3* gene, present in Combo-1 panel is overexpressed in early stage and is a DNase I-family endonuclease that has previously been associated in inducing apoptosis in cancer cell lines^26^,^27^,^28^. Another marker *BMP5*, present in Combo-1 panel has also been implicated in pancreatic and other cancers and has shown lower expression in cancer cells as compared to normal cells, indicating that expression of *BMP5* decreases in the later stages^29^. *FRMPD2*, present in Combo-2 panel has shown potential role in tight junction formation and also known to be downregulated in various epithelial cancer cell lines^30^. Likewise, in our study, this gene is found to be downregulated in the late stage as compared to early stages of ccRCC. The Combo-1 and Combo-2 panels provide a few genes that already have their implication as putative biomarkers in other cancers and also suggest some novel genes like *ENAM*, whose role in cancer has not been investigated.

After analyzing the data using simple threshold based methods, we used well-known feature selection methods to get a list of putative biomarkers. The final set of 64 genes (RCSP-set-Weka) is selected by resampling 100 times and has maximum overlap among all the 100 sets. It evidently discriminated with fair ROC of 0.81 on the external validation dataset. Next, the biomarker panel is selected only from cancer Hallmark GO terms. With 38 gene subset (RCSP-set-Weka-Hall), ROC of 0.78 was obtained on the validation dataset. The integration of cancer hallmark GO-term feature selection module in the server is an advancement in the study by reducing the number of features substantially without loosing the performance. Resampling of the dataset also facilitated the robustness of proposed ccRCC marker genes. Many of the biomarkers found in our study have already been implicated in renal and other cancers (Supplementary Table S7).

In this study, we have also attempted to develop gender-specific models for male and female datasets. The different genes selected as putative biomarkers throw insights on the differential regulation and development of tumor-related genes in males and females. The main lead in this study is that we have been able to achieve reasonable performance on very less number of genes that could be used for predicting the stage of renal cancer. We have also developed a web-based platform CancerCSP, which can analyze the gene expression data of a sample and predict whether it is an early stage patient or late stage patient with a score using RSEM values.

## Conclusion

In brief, this study categorizes early and late stage patients of ccRCC, using gene expression data with simple threshold-based classification methods and general machine learning techniques. The feature space has been effectively condensed from nearly 20,000 genes to minimum eight genes using simple threshold-based models. The Combo-1 set based threshold models achieved ROC 0.77 with accuracy 70.19%. The features selected through Weka using correlation-based algorithm provided 64 features, (RCSP-set-Weka) which gave 72.64% accuracy and ROC of 0.81 on the validation dataset. We also developed RCSP-set-Weka-Hall subset consisting of 38 genes, selecting only from subset of genes associated with cancer hallmark processes. This set gave similar performance to RCSP-set-Weka with lesser number of genes and thus gives major putative markers which can help in asserting progression of cancer to late stage. We have also tried to develop gender-specific models that could increase the performance of prediction in a particular gender.

Ultimately, a web platform CancerCSP is developed where the user can provide gene expression (RSEM values) and can predict whether the cancer is in the early or late stage. This type of machine learning application where minimum numbers of genes are used to delineate the early and late stage of cancer using high-throughput data can provide better insights to understand the mechanisms responsible for metastasis in various cancers. The study possesses clinical as well as prognostic potential by predicting the stage of ccRCC patients. Hence, the resource will be of utmost use for biological researchers and even medical practitioners for making preliminary postulations regarding cancer staging.

## Methods

### Datasets

The Level 3 RNAseq expression data for 523 ccRCC patients with KIRC (Kidney Renal Clear Cell Carcinoma) was obtained from the TCGA data portal with their clinical information in the form of Biospecimen Core Resource (BCR) IDs for patients from the Biotab utility. The dataset provides gene expression values in the form of RSEM for 20,531 genes in tumor samples of the patients. The obtained data consists of raw counts and RSEM values. In our work, RSEM values were used as quantification values, and only tumor samples with matched normal or unmatched normal were taken into consideration. We defined stage I and stage II patients as early stage patients and stage III and stage IV patients as late stage patients. In this study, 80% samples (419 patients) were used for training and testing called training dataset. Remaining 20% samples (104 patients) were used for external validation called as independent or external validation dataset (Supplementary Table S8). The other general clinical characteristics like age and gender are shown in Supplementary Figure S6.

### Processing of Data

It was observed that gene expression values have a wide range of variation; therefore we transformed the RSEM values using log_2_ after adding 1.0 as a constant number to each RSEM value. Before normalizing the data, we removed the features with low variance of 0.25 using *caret* package in R^31^. After removing low variance features, the features were reduced to 19,166 from 20,531. Subsequently, we normalized the log_2_ transformed RSEM values for each gene and converted it to z-score using the *caret* R package. Following equations were used for computing the transformation and normalization:


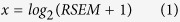



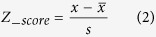


Where *Z_*_*score*_ is the normalized score, *x* is the log-transformed gene expression, 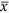 is the mean expression of a gene in the training dataset, and *s* is the standard deviation of a gene in the training dataset. The mean and standard deviation of training features were used to normalize the validation dataset.

### Development of prediction models

#### Threshold-based model

In this study, a simple approach has been used to discriminate early and late stage of cancer using the expression of a gene. This method is based on the fact that few genes are differentially expressed in different stages of cancer. In this approach, for every gene, we selected a threshold, which determines whether a sample is in an early or late stage according to the expression of that gene. If a gene is overexpressed in the early stage *i.e*. its average normalized expression is more in early stage as compared to the late stage in training data and for a given sample its normalized expression is more than the threshold, then we classify that sample as early stage otherwise as late stage. Whereas, if the gene is overexpressed in late stage *i.e*. its average normalized expression is less in the early stage as compared to the late stage in training data and for a given sample its normalized expression is more than the threshold, then we classify that sample as late stage otherwise as early stage. Further, to optimize the threshold to achieve the best performance, iteration technique was used; where the threshold was increased or decreased systematically for a range of normalized expression values across all the samples for a particular gene. For every gene, that threshold was selected, which gave maximum classification performance in terms of area under receiver operating characteristic curve (ROC). In this study, we called this approach as threshold approach and these models as threshold-based models.

#### Implementation of machine learning techniques

In order to develop machine learning based models, we used two software packages SVM^*light*^ 32 and Weka^33^. Here we used RBF kernel of SVM at different parameters; g ∈ [10^−4^–10], c ∈ [1, 2, 3, 4, 5, 6, 7, 8, 9, 10], j ∈ [1, 2, 3, 4, 5] for optimizing the SVM performance. Random forests, SMO, Naïve Bayes, J48 were implemented using Weka software.

#### Feature Selection

Feature selection is a major step for selecting the relevant features across a large number of features for developing better classification models. This selection also rules out the possibility of overfitting in prediction models. In this study, expression of genes is used as features for classification of samples. These features or genes were further ranked or selected using following techniques.

#### Features selection using threshold-based models

In order to rank the genes, we developed a threshold-based model for each gene and computed the discriminatory power of the model in terms of ROC. It means the discriminatory power of a gene is proportional to the performance (ROC) of the threshold-based model of a gene. We ranked all the genes based on ROC value of their model and selected top 50 genes having maximum discriminatory power. For eliminating the redundancy among genes or features, we removed all those genes having correlation 0.60 or more. After removing the redundant genes, we got a set of 28 genes where no two genes had correlation more than 0.60. In addition to individual gene-based models, we also developed threshold-based models using two or more genes. In this case, we computed mean expression of a number of genes and developed a threshold-based model using the mean expression of these genes. Following equation is used for computing the mean expression of genes.


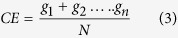


where *CE* is the mean expression, *N* is number of genes, *g*_*1*_ is normalized gene expression of gene *G*_*1*_; *g*_*n*_ is normalized gene expression of gene *Gn*.

It is important to note that this technique works only when we combine genes of one type (e.g., overexpressed or underexpressed in the early stage of cancer). We cannot combine genes where some genes are overexpressed in the early stage of cancer and remaining are overexpressed in late stage of cancer.

#### Feature selection for machine learning techniques

Ranking genes based on SVM models: In this technique, first we selected the best gene called G1, showing highest discriminatory power in threshold-based models. In next step, we combine gene G1 iteratively with other genes and further identify the best gene, which provides the best performance with gene G1. The gene that gives the best performance with G1 is called gene G2. This means two-genes based SVM model gives the best performance using gene G1 and G2. Similarly, we developed three genes based SVM model where we identified the third gene G3 that gives the best performance with G1 and G2. This process is repeated to rank the genes.

Weka-Based: In addition, we selected features using software package Weka^34^ and used attribute evaluator named, ‘SymmetricalUncertAttributeSetEval’ with search method of ‘FCBFSearch’. The algorithm Fast Correlation-Based Feature (FCBF) selection utilizes predominant correlation to identify relevant features in high-dimensional datasets in reduced feature space^35^. In order to select the robust features, the data was split into the ratio of 80:20 for 100 times followed by features selection using Weka every time on the training dataset. From this resampling process, we obtained 100 sub-sets of features. The feature sub-set depicting maximum overlap in terms of number of features with other sub-sets was selected for model development.

Weka-hallmark based: In order to identify key genes involved in ccRCC, we selected all the genes from cancer hallmark processes given by Hanahan and Weinberg^36^. They defined the multistep development of human tumors with acquisition of six biological capabilities which include sustaining proliferative signaling, escaping growth suppressors, defying cell death, supporting replicative immortality, inducing angiogenesis and metastasis, reprogramming of energy metabolism and evading immune destruction. Many earlier studies have used cancer hallmark genes to define the genes responsible for prognosis of cancer^37^,^38^. Out of the total 20,000 genes 4,843 genes mapped to the cancer hallmark GO terms. To further select genes from already known cancer related genes, we did feature selection only using these genes and for 9 hallmark processes.

### Gender-Specific Features

We have also developed gender-specific models by separating 338 males and 185 females (ccRCC patients) from the TCGA samples. Here, training and validation data have been used to develop 10-fold cross-validated models for males and females. Features were selected on the basis of above described Weka algorithm and threshold-based ROC calculation.

### Cross-validation technique

The 10-fold cross-validation technique was used to evaluate the performance of various SVM models. In this technique, patients were indiscriminately separated into ten sets, of which nine sets were used for training and the remaining set was used for testing. The process is recapped ten times in such a way that each set is used just once for validation. The final performance was obtained by taking the mean performance of all the ten sets.

### Performance measures

The performance of various models developed in this study was computed using threshold-dependent as well as threshold-independent parameters. In threshold-dependent parameters, we used sensitivity (Sn), specificity (Sp), overall accuracy (Ac) and Matthews correlation coefficient (MCC) using the following equations:


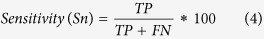



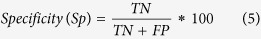



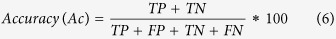



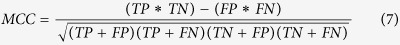


where TP and TN are the true positive and true negative predictions. FP and FN are false positive and false negative predictions respectively.

The above parameters are threshold dependent parameters for measuring the performance of a model. It means performance measures described above will vary with the threshold. Thus, it is difficult to measure the performance of a model using single parameter. In order to overcome this problem, we also calculated a threshold-independent parameter called ROC, which is computed from receiver operating characteristic (ROC) plot in this study. The ROC curve is created by plotting sensitivity or true positive rate against the false positive rate (1-specificity) at different thresholds. Finally, we calculated the area under ROC curve to compute a single parameter from this curve called ROC in this study. We used this ROC value for optimizing and measuring the performance of our models.

### Gene annotation and enrichment analysis

In order to estimate the efficiency and biological implications of these predicted markers in ccRCC, the genes were subjected to functional enrichment analyses. Various biological parameters including their molecular functions, biological processes, regulatory elements and cellular components were identified through intensive manual curation and a web-based gene set analysis toolkit (WebGestalt)^39^. The enrichment was characterized via *p*-values using hypergeometric test, further adjusted by Benjamini and Hochberg’s multiple testing approach. The markers obtained from all the datasets were then analyzed for interaction studies where the association among these markers and other regulatory elements were elucidated using KEGG^40^ and STRING databases^41^ providing clues for their putative roles in ccRCC.

## Additional Information

**How to cite this article:** Bhalla, S. *et al*. Gene expression-based biomarkers for discriminating early and late stage of clear cell renal cancer. *Sci. Rep.* **7**, 44997; doi: 10.1038/srep44997 (2017).

**Publisher's note:** Springer Nature remains neutral with regard to jurisdictional claims in published maps and institutional affiliations.

## Supplementary Material

Supplementary Information

## Figures and Tables

**Table 1:** The performance of single gene-based threshold models developed using top overexpressed and under-expressed genes in early stage of ccRCC patients along with the brief description of molecular function and cancer hallmark biological process (Cancer hallmark GO term) associated with each gene.

S. No.	Gene	Threshold	Performance		Molecular Function	Cancer hallmark GO terms
			Accuracy (%)	ROC		
***Overexpressed genes***						
1	*NR3C2*	−0.48	71.12	0.67	ACE Inhibitor Pathway, Aldosterone-regulated sodium reabsorption, transcription factor activity, sequence-specific DNA binding, steroid hormone receptor activity	—
2	*C1orf69*	−0.04	66.83	0.67	Transferase activity, poly(A) RNA-binding	—
3	*FAM160A1*	0.06	65.39	0.66	—	—
4	*FRMPD2*	0.09	63.72	0.65	1-phosphatidylinositol binding	—
5	*BMP5*	−0.01	63.01	0.65	Induces bone and cartilage development	—
6	*TSPYL4*	0.18	63.48	0.65	Nucleosome assembly	—
7	*SLC30A9*	−0.22	66.59	0.65	Transcription factor activity, sequence-specific DNA binding, cationtransmembrane transporter activity, ligand-dependent nuclear receptor binding	DNA repair
8	*FAM122A*	0.23	62.77	0.64	—	—
9	*DNASE1L3*	0.27	61.58	0.64	Cleaves chromatin DNA to nucleosomal units, endonuclease activity, calcium ion binding	—
10	*FAM190A*	−0.1	65.63	0.64	Involved in cell division	Cell cycle
***Underexpressed genes***						
11	*PLEKHA9*	0.48	69.93	0.65	Glycolipid binding, glycolipid transporter activity	—
12	*SMPD4*	0.2	67.54	0.65	Sphingolipid metabolic and catabolic process	—
13	*AGTR1*	−0.19	66.35	0.65	G-protein coupled receptor activity, bradykinin receptor binding, angiotensin type I and type II receptor activity	Cell motility, Response to external stimulus
14	*TMEM214*	−0.37	61.58	0.65	Mediates endoplasmic reticulum (ER) stress-induced apoptosis by activating CASP4	—
15	*CCM2*	0.16	66.11	0.65	Crucial regulator of heart and vessel formation and integrity	Phosphorylation
16	*MAP6D1*	0.05	66.35	0.65	Calmodulin binding, microtubule binding	—
17	*SESTD1*	−0.37	68.02	0.65	Phosphatidylinositol-4,5-bisphosphate binding, phosphatidic acid binding	—
18	*CASP4*	0.2	65.39	0.65	Mediates endoplasmic reticulum (ER) stress-induced apoptosis, cysteine-type peptidase and endopeptidase activity	Apoptosis, Immune response
19	*IRF7*	0.38	67.06	0.65	Transcription factor activity, RNA polymerase II core promoter proximal region sequence-specific binding	Immune response, Response to external stimulus
20	*LPAR2*	0.32	66.35	0.64	G-protein coupled receptor activity, PDZ domain binding, Stimulates phospholipase C	—

**Table 2:** The performance of classification models based on RCSP-set-Threshold (28 genes) developed using different machine learning techniques on training and independent or external validation dataset.

Technique	Dataset	Performance Measures				
		Sensitivity	Specificity	Accuracy (%)	MCC	ROC
RF	Training	73.62	72.12	73.03	0.45	0.77
	Validation	73.02	60.98	68.27	0.34	0.74
Naive Bayes	Training	75.98	67.27	72.55	0.43	0.76
	Validation	77.78	60.98	71.15	0.39	0.76
SMO	Training	83.86	55.76	72.79	0.42	0.70
	Validation	80.95	53.66	70.19	0.36	0.67
J48	Training	64.17	66.06	64.92	0.3	0.67
	Validation	68.25	58.54	64.42	0.26	0.67
SVM	Training	75.98	69.09	73.27	0.45	0.78
	Validation	74.6	65.85	71.15	0.4	0.77

These RCSP-set-Threshold features are selected by the threshold-based approach followed by the removal of correlated features.

**Table 3:** The performance of Support vector machine (SVM) and Random Forest (RF) based models developed using different sets of selected features on training and independent or external validation dataset.

Features	Dataset	Technique	Performance Measures				
			Sensitivity	Specificity	Accuracy (%)	MCC	ROC
setA-1 (4 genes)	Training	SVM	71.65	70.3	71.12	0.41	0.76
	Validation		68.25	78.05	72.12	0.45	0.80
	Training	RF	70.87	65.45	68.74	0.36	0.69
	Validation		73.02	58.54	67.31	0.32	0.74
setB-1 (4 genes)	Training	SVM	71.26	70.3	70.88	0.41	0.74
	Validation		74.6	68.29	72.12	0.42	0.74
	Training	RF	80.31	49.7	68.26	0.32	0.65
	Validation		82.54	51.22	70.19	0.36	0.68
Combo-1 (8 genes)	Training	SVM	75.20	70.30	73.27	0.45	0.77
	Validation		77.78	68.29	74.04	0.46	0.80
	Training	RF	81.1	55.15	70.88	0.38	0.73
	Validation		82.54	51.22	70.19	0.36	0.74

These gene sets include setA-1 (4 overexpressed genes), setB-1 (4 under-expressed genes) and Combo-1 (combination of both gene sets *i.e*. setA-1 and setB-1).

**Table 4:** The performance of Support vector machine (SVM) and Random Forest (RF) models developed using different sets of features selected via SVM technique on training and independent or external validation dataset.

Features	Dataset	Technique	Performance Measures				
			Sensitivity	Specificity	Accuracy (%)	MCC	ROC
setA-2 (5 genes)	Training	SVM	68.9	73.94	70.88	0.42	0.75
	Validation		65.08	70.73	67.31	0.35	0.75
	Training	RF	81.5	56.97	71.84	0.4	0.73
	Validation		77.78	46.34	65.38	0.25	0.68
setB-2 (5 genes)	Training	SVM	68.9	70.91	69.69	0.39	0.76
	Validation		60.32	70.73	64.42	0.3	0.72
	Training	RF	71.65	64.85	68.97	0.36	0.71
	Validation		69.84	56.1	64.42	0.26	0.70
Combo-2 (10 genes)	Training	SVM	72.44	72.89	72.62	0.45	0.78
	Validation		71.43	68.29	70.19	0.39	0.77
	Training	RF	76.19	65.85	72.12	0.42	0.76
	Validation		70.47	70.3	70.41	0.4	0.76

**Table 5:** The performance of models based on different machine techniques using RCSP-set-Weka (64 genes) selected by Weka.

Technique	Dataset	Performance Measures				
		Sensitivity	Specificity	Accuracy (%)	MCC	ROC
RF	Training	80.63	81.10	80.82	0.61	0.87
	Validation	78.12	64.29	72.64	0.43	0.75
Naive Bayes	Training	79.05	70.12	75.54	0.49	0.81
	Validation	75.00	64.29	70.75	0.39	0.76
SMO	Training	84.19	70.12	78.66	0.55	0.77
	Validation	79.69	66.67	74.53	0.47	0.73
J48	Training	67.19	74.39	70.02	0.41	0.73
	Validation	64.06	88.10	73.58	0.51	0.79
SVM	Training	79.84	75.61	78.18	0.55	0.83
	Validation	73.44	71.43	72.64	0.44	0.81

These models were evaluated using 10-fold cross validation on training dataset as well as on independent or external validation dataset.

**Table 6:** The performance of models developed using different machine techniques based on RCSP-set-Weka-Hall (38 genes) selected from Weka.

Technique	Dataset	Performance Measures				
		Sensitivity	Specificity	Accuracy (%)	MCC	ROC
RF	Training	75.10	78.66	76.50	0.53	0.84
	Validation	67.19	78.57	71.70	0.45	0.75
Naive Bayes	Training	79.84	71.34	76.50	0.51	0.83
	Validation	75.00	66.67	71.70	0.41	0.79
SMO	Training	85.77	66.46	78.18	0.54	0.76
	Validation	82.81	59.52	73.58	0.44	0.71
J48	Training	71.54	61.59	67.63	0.33	0.69
	Validation	68.75	71.43	69.81	0.39	0.68
SVM	Training	80.24	73.78	77.70	0.54	0.83
	Validation	73.44	71.43	72.64	0.44	0.78

These genes are specifically involved in cancer hallmark biological processes (Cancer hallmark GO terms). The model was evaluated using 10-fold cross validation on training dataset as well as on independent external validation dataset.

**Table 7:** The performance of gender-specific Support vector machine (SVM) and Random Forest (RF) models developed using Weka selected genes/features on training and independent or external validation dataset.

Gender	Technique	Dataset	Performance Measures				
			Sensitivity	Specificity	Accuracy (%)	MCC	ROC
Female	RF	Training	87.1	88.89	87.76	0.75	0.93
		Validation	75	71.43	73.68	0.45	0.76
	SVM	Training	89.25	79.63	85.71	0.69	0.90
		Validation	75	85.71	78.95	0.59	0.82
Male	RF	Training	83.02	73.39	79.10	0.57	0.83
		Validation	75.61	58.62	68.57	0.35	0.72
	SVM	Training	83.02	76.15	80.22	0.59	0.87
		Validation	78.05	75.86	77.14	0.53	0.80
