# Supplementary material for: Gene expression-based biomarkers for discriminating early and late stage of clear cell renal cancer
Source: Sci Rep. 2017 Mar 28;7:44997. doi: 10.1038/srep44997 (PMC5368637; doi:10.1038/srep44997)
Supplement: Supplementary Information [file srep44997-s1.doc]

**Supplementary Information File**

**Gene expression-based biomarkers for discriminating early and late stage of clear cell renal cancer**

Sherry Bhalla^1*^, Kumardeep Chaudhary^1*^,Ritesh Kumar^2^, Manika Sehgal^1^, Harpreet Kaur^1^, Suresh Sharma^3^, Gajendra P.S. Raghava^1#^

^1^Bioinformatics Centre, CSIR-Institute of Microbial Technology, Sector 39A, Chandigarh-160036, India

^2^CSIR-Central Scientific Instruments Organization, Sector 30C, Chandigarh-160030, India

^3^Centre for Systems Biology and Bioinformatics, Panjab University, Sector 14, Chandigarh-160014, India

*Joint First Authors

**#Address for correspondence**

G.P.S. Raghava

Email: [raghava@imtech.res.in](mailto:raghava@imtech.res.in)

Web: <http://www.imtech.res.in/raghava/>

Phone: +91-172-2690557

Fax: +91-172-2690632

**Table S1.**The performance of threshold-based models developed using over and under expressed genes (top 1, 2, 3 and 4 genes).

| **Genes overexpressed in early stage** | **Performance Measures** | | |
| --- | --- | --- | --- |
|  | **Threshold** | **Accuracy (%)** | **ROC** |
| *NR3C2* | -0.480 | 71.12 | 0.67 |
| *NR3C2, ENAM* | -1.179 | 72.08 | 0.70 |
| *NR3C2, ENAM, DNASE1L3* | -0.290 | 72.55 | 0.69 |
| *NR3C2, ENAM, DNASE1L3, FRMPD2* | 0.120 | 70.17 | 0.71 |
| **Genes overexpressed in late stage** | **Threshold** | **Accuracy (%)** | **ROC** |
| *PLEKHA9* | 0.480 | 69.93 | 0.65 |
| *PLEKHA9, MAP6D1* | 0.127 | 71.36 | 0.69 |
| *PLEKHA9, MAP6D1, SMPD4* | -0.018 | 70.88 | 0.70 |
| *PLEKHA9, MAP6D1, SMPD4, C11orf73* | 0.135 | 73.03 | 0.71 |

**Table S2. The performance of SVM-based models developed using overexpressed and underexpressed genes (1, 2, 3 and 4 genes). These features were selected using SVM.**

| **Genes overexpressed in early stage** | **Performance Measures** | | |
| --- | --- | --- | --- |
|  | **Threshold** | **Accuracy (%)** | **ROC** |
| *NR3C2* | 0.6 | 69.93 | 0.73 |
| *NR3C2,C1orf69* | 0.6 | 70.41 | 0.74 |
| *NR3C2,C1orf69,BMP5* | 0.3 | 71.12 | 0.75 |
| *NR3C2,C1orf69,BMP5,DNASE1L3* | 0.4 | 71.84 | 0.76 |
| *NR3C2, C1orf69, BMP5, DNASE1L3, NBEA* | 0.4 | 72.32 | 0.77 |
| **Genes overexpressed in Late stage** | **Threshold** | **Accuracy (%)** | **ROC** |
| *PLEKHA9* | 0.8 | 64.68 | 0.70 |
| *PLEKHA9, IRF7* | 0.8 | 65.45 | 0.73 |
| *PLEKHA9, IRF7, TMEM214* | 0.6 | 68.97 | 0.74 |
| *PLEKHA9, IRF7, TMEM214, PITX1* | 0.7 | 68.74 | 0.75 |
| *PLEKHA9, IRF7, TMEM214, PITX1, PLA2G2A* | 0.7 | 69.45 | 0.76 |

**Table S3.** The pathways and cancer hallmark GO terms associated with RCSP-Weka-Hall set of putative biomarkers.

| **S. No.** | **Gene** | **Gene Details** | **Pathways** | **Hallmark GO terms** |
| --- | --- | --- | --- | --- |
| 1 | *SPOCK1* | Sparc/osteonectin, cwcv and kazal-like domains proteoglycan (testican) 1 | MAPK signaling pathway, Integrin pathway, PTEN pathway, Adipogenesis | Cell motility |
| 2 | *EMX2* | Empty spiracles homeobox 2 | Wnt signaling pathways | Cell motility |
| 3 | *TLR9* | toll-like receptor 9 | Malaria, Chagas disease (American trypanosomiasis) | Immune response, Phosphorylation, Response to external stimulus |
| 4 | *MNX1* | motor neuron and pancreas homeobox 1 | Neural Stem Cell Differentiation | Immune response |
| 5 | *SEMA3G* | sema domain, immunoglobulin domain (Ig), short basic domain, secreted, (semaphorin) 3G | Axon guidance | Response to external stimulus, Cell motility |
| 6 | *GPR77* | G protein-coupled receptor 77 | Signaling by GPCR | Response to external stimulus |
| 7 | *COX7B* | cytochrome c oxidase subunit VIIb | Respiratory electron transport | Phosphorylation |
| 8 | *NODAL* | nodal homolog (mouse) | TGF-beta signaling pathway | Cell motility |
| 9 | *TNFSF4* | tumor necrosis factor (ligand) superfamily, member 4 | Cytokine-cytokine receptor interaction | Immune response, Response to external stimulus |
| 10 | *UBE2D3* | ubiquitin-conjugating enzyme E2D | Ubiquitin-Proteasome Dependent Proteolysis | DNA repair |
| 11 | *PIP5K1B* | phosphatidylinositol-4-phosphate 5-kinase, type I, beta | Endocytosis | Phosphorylation |
| 12 | *NEIL1* | nei endonuclease VIII-like 1 (E. coli) | Base excision repair | DNA repair |
| 13 | *HUS1B* | HUS1 checkpoint homolog b (S. pombe) | p53 signaling pathway | Cell cycle, DNA repair |
| 14 | *POLD3* | polymerase (DNA-directed), delta 3, accessory subunit | Base excision repair | DNA repair |
| 15 | *RPL39* | ribosomal protein L39 | Transport to the Golgi and subsequent modification | Immune response, Response to external stimulus |
| 16 | *CTSG* | cathepsin G | Peptide ligand-binding receptors | Immune response, Phosphorylation, Response to external stimulus |
| 17 | *DSC2* | desmocollin 2 | cell adhesion | Response to external stimulus |
| 18 | *MAP3K13* | mitogen-activated protein kinase kinase kinase 13 | MAPK signaling pathway | Phosphorylation |
| 19 | *FUT10* | fucosyltransferase 10 (alpha (1,3) fucosyltransferase) | Protein modification | Cell motility |
| 20 | *IL10RB* | interleukin 10 receptor, beta | Cytokine-cytokine receptor interaction | Immune response, Response to external stimulus |
| 21 | *CLDN7* | claudin 7 | Cell junstion organization, cell adhesion molecules (CAMs) | Cell Adhesion, Response to external stimulus |
| 22 | *SCG2* | secretogranin II | MAPK cascade, Angiogenesis | Phosphorylation, Response to external stimulus, Cell motility |
| 23 | *FGFR3* | fibroblast growth factor receptor 3 | Endocytosis, MAPK signaling pathway, Pathways in cancer | Phosphorylation |
| 24 | *TGFB3* | transforming growth factor, beta 3 | Cytokine-cytokine receptor interaction, Endocytosis, Malaria, MAPK signaling pathway, Rheumatoid arthritis, Pathways in cancer, TGF-beta signaling pathway, Chagas disease (American trypanosomiasis) | Cell growth |
| 25 | *CXCL5* | chemokine (C-X-C motif) ligand 5 | Cytokine-cytokine receptor interaction, Rheumatoid arthritis | Immune response, Response to external stimulus, Cell motility |
| 26 | *ADM* | adrenomedullin | GPCR signaling | Immune response, Response to external stimulus |
| 27 | *PGLYRP2* | peptidoglycan recognition protein 2 | Signal transduction | Immune response, Response to external stimulus |
| 28 | *BEST1* | bestrophin 1 | Ion channel transport | Response to external stimulus |
| 29 | *ZNF830* | zinc finger protein 830 | Nucleotide excision repair | Cell cycle, DNA repair |
| 30 | *SOX9* | SRY (sex determining region Y)-box 9 | Wnt signaling, cAMP signaling | Phosphorylation, Response to external stimulus, Cell growth |
| 31 | *SELS* | Selenoprotein S | ER stress pathway | Response to external stimulus |
| 32 | *NKX2-3* | NK2 homeobox 3 | Plasma cell differentiation | Immune response, Cell motility |
| 33 | *STK38* | serine/threonine kinase 38 | MAPK patway, Signal transduction | Phosphorylation |
| 34 | *GHSR* | growth hormone secretagogue receptor | GPCR ligand binding, CREB pathway, cAMP signaling | Response to external stimulus |
| 35 | *RXRA* | Retinoid X receptor, alpha | PPAR signaling pathway, Pathways in cancer | Response to external stimulus |
| 36 | *FABP7* | fatty acid binding protein 7, brain | PPAR signaling pathway | Response to external stimulus |
| 37 | *SLC22A16* | Solute carrier family 22 (organic cation/carnitine transporter), member 16 | Organic cation transport,  Transmembrane transport | Cell motility |
| 38 | *SGK223* | homolog of rat pragma of Rnd2 | JAK1/Stat3 signaling | Phosphorylation |

**Table S4.** The performance of the SVM classification models based on different types of feature sets and their combinations.

| **Features/Combinations** | **Dataset** | **Sensitivity** | **Specificity** | **Accuracy (%)** | **MCC** | **ROC** |
| --- | --- | --- | --- | --- | --- | --- |
| RCSP-set-Weka  (A) | Training Data | 79.84 | 75.61 | 78.18 | 0.55 | 0.83 |
|  | Validation Data | 73.44 | 71.43 | 72.64 | 0.44 | 0.81 |
| RCSP-set-Hall-Weka  (B) | Training Data | 80.24 | 73.78 | 77.70 | 0.54 | 0.83 |
|  | Validation Data | 73.44 | 71.43 | 72.64 | 0.44 | 0.78 |
| RCSP-set-Threshold  (C) | Training Data | 75.98 | 69.09 | 73.27 | 0.45 | 0.78 |
|  | Validation Data | 74.6 | 65.85 | 71.15 | 0.40 | 0.77 |
| 96 features  (A + B) | Training Data | 75 | 76.19 | 75.47 | 0.50 | 0.85 |
|  | Validation Data | 77.08 | 75.61 | 76.5 | 0.52 | 0.83 |
| 66 Features  (B + C) | Training Data | 81.42 | 71.34 | 77.46 | 0.53 | 0.81 |
|  | Validation Data | 84.38 | 71.43 | 79.25 | 0.56 | 0.84 |
| 90 Features  (A + C) | Training Data | 73.12 | 81.71 | 76.5 | 0.54 | 0.83 |
|  | Validation Data | 68.75 | 73.81 | 70.75 | 0.42 | 0.82 |
| 122 Features  (A + B + C) | Training Data | 72.73 | 82.32 | 76.5 | 0.54 | 0.84 |
|  | Validation Data | 70.31 | 78.57 | 73.58 | 0.48 | 0.84 |

**Table S5.** Comparison of top 10 genes in males, showing maximum difference in their average gene expression as compared to females.

| **Gene** | **Male** | | | | **Female** | | | |
| --- | --- | --- | --- | --- | --- | --- | --- | --- |
|  | **Average Gene Expression** | | **Difference** | **ROC** | **Average Gene Expression** | | **Difference** | **ROC** |
|  | **Early Stage** | **Late Stage** |  |  | **Early Stage** | **Late Stage** |  |  |
| *SLC6A19* | 7.02 | 4.86 | 2.16 | 0.63 | 9.08 | 6.61 | 2.47 | 0.65 |
| *PLG* | 4.88 | 3.1 | 1.78 | 0.6 | 5.42 | 3.01 | 2.41 | 0.64 |
| *SLC5A8* | 7.32 | 5.59 | 1.73 | 0.62 | 8.83 | 7.11 | 1.72 | 0.61 |
| *G6PC* | 5.57 | 4.04 | 1.54 | 0.64 | 6.14 | 4.91 | 1.23 | 0.61 |
| *FTCD* | 7.19 | 5.72 | 1.47 | 0.61 | 7.43 | 7.07 | 0.35 | 0.52 |
| *TMEM174* | 6.65 | 5.21 | 1.44 | 0.58 | 7.52 | 6.04 | 1.47 | 0.61 |
| *LOC388387* | 6.16 | 4.75 | 1.4 | 0.6 | 7.11 | 6.44 | 0.67 | 0.57 |
| *UNC5D* | 3.65 | 2.26 | 1.38 | 0.63 | 4.85 | 3.73 | 1.13 | 0.59 |
| *PCK1* | 9.99 | 8.62 | 1.38 | 0.57 | 10.78 | 9.01 | 1.77 | 0.64 |
| *ENAM* | 6.57 | 5.21 | 1.36 | 0.65 | 7.2 | 5.83 | 1.36 | 0.65 |

**Table S6.** Comparison of top 10 genes in females, showing maximum difference in their average gene expression as compared to males.

| **Gene** | **Female** | | | | **Male** | | | |
| --- | --- | --- | --- | --- | --- | --- | --- | --- |
|  | **Average Gene Expression** | | **Difference** | **ROC** | **Average Gene Expression** | | **Difference** | **ROC** |
|  | **Early Stage** | **Late Stage** |  |  | **Early Stage** | **Late Stage** |  |  |
| *SLC6A19* | 9.08 | 6.61 | 2.47 | 0.65 | 7.02 | 4.86 | 2.16 | 0.63 |
| *PLG* | 5.42 | 3.01 | 2.41 | 0.64 | 4.88 | 3.10 | 1.78 | 0.60 |
| *SOSTDC1* | 5.18 | 3.06 | 2.12 | 0.70 | 4.39 | 3.84 | 0.55 | 0.56 |
| *CA4* | 7.31 | 5.38 | 1.92 | 0.67 | 6.16 | 5.01 | 1.15 | 0.59 |
| *LOC283392* | 6.23 | 4.33 | 1.90 | 0.68 | 5.95 | 5.09 | 0.85 | 0.58 |
| *TCL6* | 5.06 | 3.17 | 1.89 | 0.65 | 3.87 | 2.60 | 1.27 | 0.60 |
| *TRHDE* | 8.20 | 6.33 | 1.87 | 0.67 | 7.86 | 6.85 | 1.01 | 0.58 |
| *SLC22A8* | 4.67 | 2.82 | 1.86 | 0.63 | 3.19 | 2.81 | 0.38 | 0.53 |
| *AGTR1* | 8.32 | 6.51 | 1.81 | 0.69 | 7.43 | 6.46 | 0.96 | 0.61 |
| *SLC22A12* | 9.31 | 7.50 | 1.80 | 0.64 | 7.28 | 6.17 | 1.11 | 0.58 |

**Table S7.** The genes from RCSP-set-Weka and RCSP-set-Weka-Hall that are implicated in renal cancer and other cancers.

|  | RCSP-set-Weka | RCSP-set-Weka-Hall |
| --- | --- | --- |
| Genes in renal cancer | OTOF^1^, TBX18 ^2^, AHRR^3,4^ , ADCYAP1 ^5,6^, SLC7A7^7^, CASP9 ^8,9^, FGF5^10,11^ ,CBX4^12^, SCNN1G^13^,SLC7A13 ^14^, FGFR3^15^, SLC22A1^16^, SLC9A3^17^ | TLR9^17^, NODAL^18^, HUS1B^19^, CLDN7^20^, FGFR3^15,21^, TGFB3^22^, CXCL5^23^, ADM^24^, FABP7^25^ |
| Genes in other cancers | EIF5B^26^, LGALS4^27^, C3orf1^28^, EYA1^29^, SLC9A3^30^, GPR68^31^, NFE2L3^32^, TEAD3^33^,  FUT10 ^34^ , PDIA2^35^, JPH2 ^36^, TOB1^37^, CACNA1D ^38^,TFAP4^39^, LOC100132354 ^40^, VPREB3^41^, THSD7A^42^ , IQCH^43^, DMKN^44^, HUS1B^45^, DNAJC6^46^, SGK223^47^, CLDN14^48^, KIRREL^49^ , LTV1^50^, ZC3H4^51^, DBX2^52^ | SPOCK1^53^, TLR9^54^, EMX2^55,56^, MNX1^57^, SEMA3G^58,59^,  COX7B^60^, NODAL^61^, TNFSF4^62^, UBE2D3^63^, PIP5K1B^64^, RPL39^65^, CTSG^66,67^, DSC2^68^, MAP3K13^69^, FUT10^70^, NEIL1^71,72^, POLD3^73^, IL10RB^74^, CLDN7^75^, SCG2^76^, FGFR3^77,78^ |

**Table S8. Distribution of ccRCC samples in different stages, as well as allocation of samples in training and testing/independent datasets.**

| **Class** | **Clinical**  **Status** | **Training**  **Dataset** | **Testing/ Validation/ Independent Dataset** |
| --- | --- | --- | --- |
| Early Stage | Stage I | 208 | 52 |
|  | Stage II | 46 | 11 |
| Late Stage | Stage III | 100 | 25 |
|  | Stage IV | 65 | 16 |

**Figure S1.**The average normalized score of 64 features selected through Weka’s algorithm.

**Figure S2.** Comparison of ROC obtained in RCSP-set-Weka and 66 features (RCSP-set-Weka set with age and Gender as features).

**Figure S3.** Comparison of ROC obtained in RCSP-set-Weka-Hall set (38 Weka selected features from cancer hallmark genes) and 40 features (RCSP-set-Weka-Hall set with Age and Gender as features).


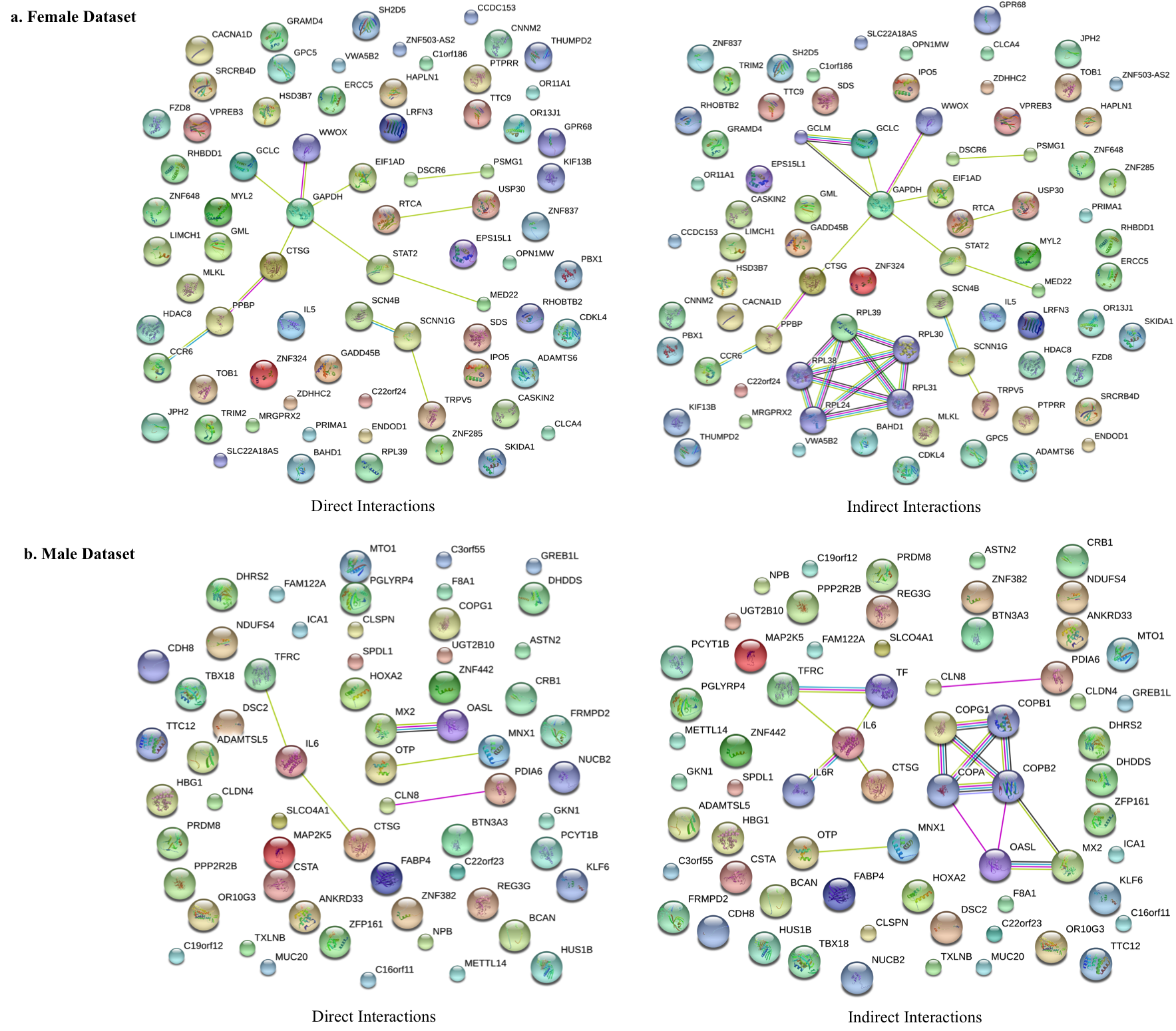


**Figure S4.** The protein-protein interaction networks (**a.** Female dataset, and **b.** Male dataset) among gender specific potential ccRCC biomarkers generated using STRING database. Adding no more than 5 interactors in first shell has generated the indirect interactions among the male and female datasets respectively.

**
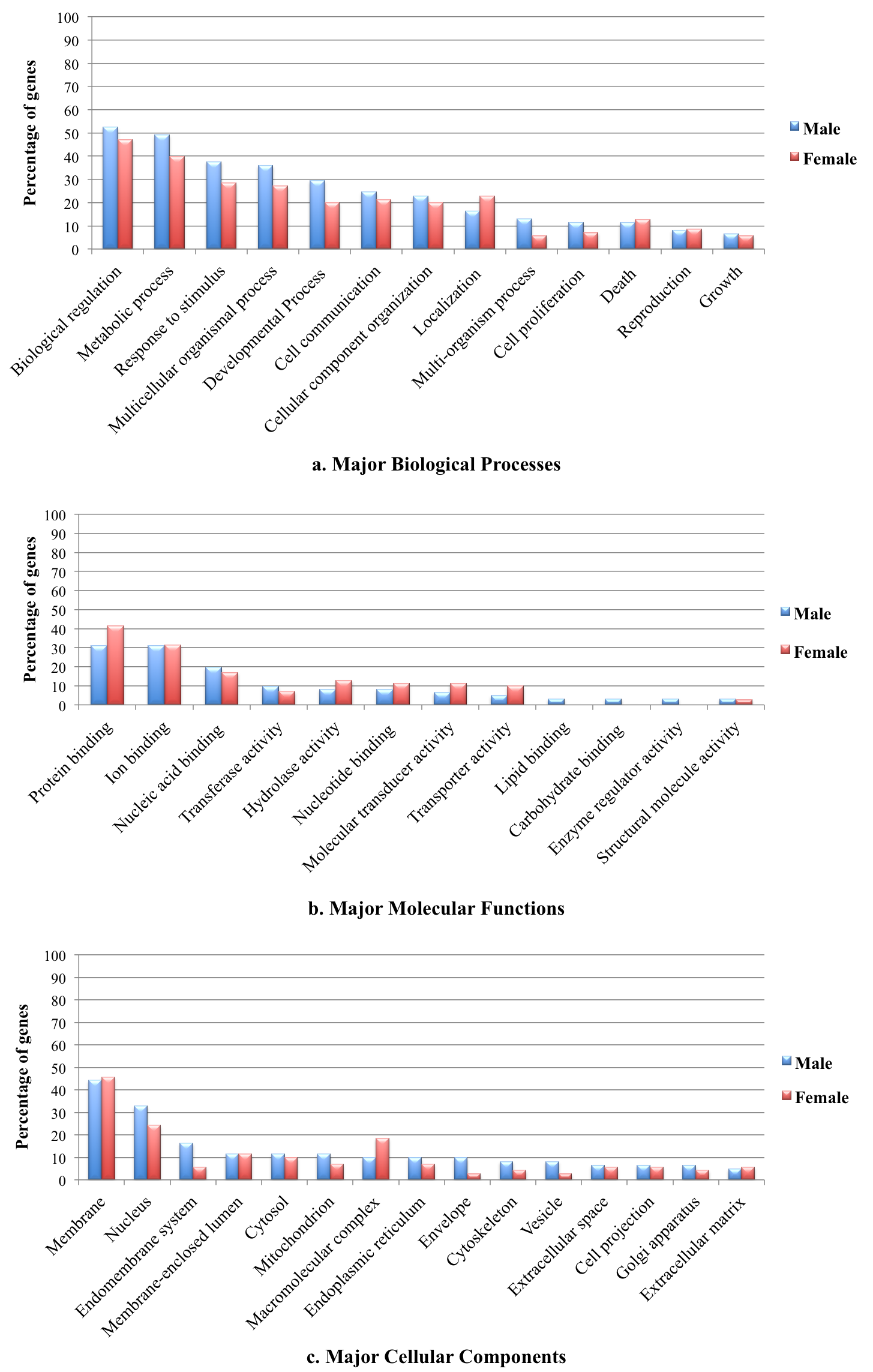
**

**Figure S5.** The gene ontology analyses depicting percentage distribution of gender specific ccRCC biomarkers in major biological processes, molecular functions and cellular components. In the process of gene enrichment, 61 out of 64 genes and 70 out of 74 genes were characterized for males and females respectively.


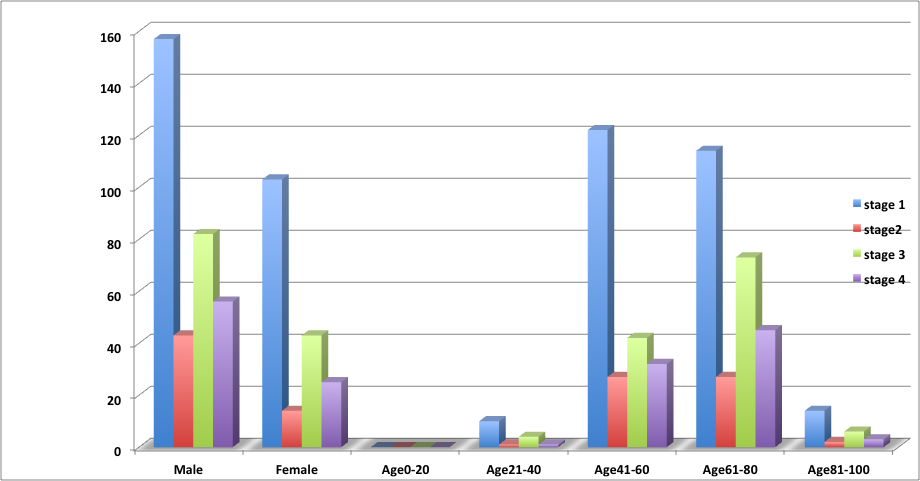


**Figure S6.** The age and gender distribution of tumor patients across various stages of cancer.

**Supplementary References**

1 Gao, S. *et al.* Identification and Construction of Combinatory Cancer Hallmark-Based Gene Signature Sets to Predict Recurrence and Chemotherapy Benefit in Stage II Colorectal Cancer. *JAMA Oncol* **2**, 37-45, (2016).

2 Kispert, A. T-Box Genes in the Kidney and Urinary Tract. *Curr Top Dev Biol* **122**, 245-278, (2017).

3 Ishida, M. *et al.* Activation of aryl hydrocarbon receptor promotes invasion of clear cell renal cell carcinoma and is associated with poor prognosis and cigarette smoke. *Int J Cancer* **137**, 299-310, (2015).

4 Harrill, J. A. *et al.* Knockout of the aryl hydrocarbon receptor results in distinct hepatic and renal phenotypes in rats and mice. *Toxicol Appl Pharmacol* **272**, 503-518, (2013).

5 Laszlo, E. *et al.* Ischemia/reperfusion-induced Kidney Injury in Heterozygous PACAP-deficient Mice. *Transplant Proc* **47**, 2210-2215, (2015).

6 Tanida, M. *et al.* Central PACAP mediates the sympathetic effects of leptin in a tissue-specific manner. *Neuroscience* **238**, 297-304, (2013).

7 Esteve, E. *et al.* Renal involvement in Lysinuric protein intolerance: contribution of pathology to assessment of heterogeneity of renal lesions. *Hum Pathol*, (2017).

8 Yuan, S. Y. *et al.* Escin induces apoptosis in human renal cancer cells through G2/M arrest and reactive oxygen species-modulated mitochondrial pathways. *Oncol Rep* **37**, 1002-1010, (2017).

9 Kedzierska, H. *et al.* Decreased Expression of SRSF2 Splicing Factor Inhibits Apoptotic Pathways in Renal Cancer. *Int J Mol Sci* **17**, (2016).

10 Hanada, K., Yewdell, J. W. & Yang, J. C. Immune recognition of a human renal cancer antigen through post-translational protein splicing. *Nature* **427**, 252-256, (2004).

11 Hanada, K., Perry-Lalley, D. M., Ohnmacht, G. A., Bettinotti, M. P. & Yang, J. C. Identification of fibroblast growth factor-5 as an overexpressed antigen in multiple human adenocarcinomas. *Cancer Res* **61**, 5511-5516, (2001).

12 de la Vega, L. *et al.* Control of nuclear HIPK2 localization and function by a SUMO interaction motif. *Biochim Biophys Acta* **1813**, 283-297, (2011).

13 Zachar, R. M. *et al.* The epithelial sodium channel gamma-subunit is processed proteolytically in human kidney. *J Am Soc Nephrol* **26**, 95-106, (2015).

14 Nagamori, S. *et al.* Novel cystine transporter in renal proximal tubule identified as a missing partner of cystinuria-related plasma membrane protein rBAT/SLC3A1. *Proc Natl Acad Sci U S A* **113**, 775-780, (2016).

15 Fiorentino, M. *et al.* Wide spetcrum mutational analysis of metastatic renal cell cancer: a retrospective next generation sequencing approach. *Oncotarget*, (2016).

16 Yang, J., Kalogerou, M., Gallacher, J., Sampson, J. R. & Shen, M. H. Renal tumours in a Tsc1+/- mouse model show epigenetic suppression of organic cation transporters Slc22a1, Slc22a2 and Slc22a3, and do not respond to metformin. *Eur J Cancer* **49**, 1479-1490, (2013).

17 Deckers, I. A. *et al.* Potential role of gene-environment interactions in ion transport mechanisms in the etiology of renal cell cancer. *Sci Rep* **6**, 34262, (2016).

18 Zhang, Z. *et al.* Nodal activates smad and extracellular signal-regulated kinases 1/2 pathways promoting renal cell carcinoma proliferation. *Mol Med Rep* **12**, 587-594, (2015).

19 Jagga, Z. & Gupta, D. Classification models for clear cell renal carcinoma stage progression, based on tumor RNAseq expression trained supervised machine learning algorithms. *BMC Proc* **8**, S2, (2014).

20 Murakami, T. *et al.* Identification and characterization of Birt-Hogg-Dube associated renal carcinoma. *J Pathol* **211**, 524-531, (2007).

21 Behbahani, T. E. *et al.* Tyrosine kinase expression profile in clear cell renal cell carcinoma. *World J Urol* **30**, 559-565, (2012).

22 Copland, J. A. *et al.* Genomic profiling identifies alterations in TGFbeta signaling through loss of TGFbeta receptor expression in human renal cell carcinogenesis and progression. *Oncogene* **22**, 8053-8062, (2003).

23 Parihar, J. S. & Tunuguntla, H. S. Role of chemokines in renal cell carcinoma. *Rev Urol* **16**, 118-121, (2014).

24 Michelsen, J. *et al.* Tissue expression and plasma levels of adrenomedullin in renal cancer patients. *Clin Sci (Lond)* **111**, 61-70, (2006).

25 Coplin, J. W. & Morgan, S. B. Learning disabilities: a multidimensional perspective. *J Learn Disabil* **21**, 614-622, (1988).

26 Holcik, M. Could the eIF2alpha-Independent Translation Be the Achilles Heel of Cancer? *Front Oncol* **5**, 264, (2015).

27 Kim, S. W. *et al.* Abrogation of galectin-4 expression promotes tumorigenesis in colorectal cancer. *Cell Oncol (Dordr)* **36**, 169-178, (2013).

28 Wu, H., Wang, W. & Xu, H. Depletion of C3orf1/TIMMDC1 inhibits migration and proliferation in 95D lung carcinoma cells. *Int J Mol Sci* **15**, 20555-20571, (2014).

29 Wu, K. *et al.* EYA1 phosphatase function is essential to drive breast cancer cell proliferation through cyclin D1. *Cancer Res* **73**, 4488-4499, (2013).

30 Kang, J. U., Koo, S. H., Kwon, K. C., Park, J. W. & Kim, J. M. Gain at chromosomal region 5p15.33, containing TERT, is the most frequent genetic event in early stages of non-small cell lung cancer. *Cancer Genet Cytogenet* **182**, 1-11, (2008).

31 Ren, J. & Zhang, L. Effects of ovarian cancer G protein coupled receptor 1 on the proliferation, migration, and adhesion of human ovarian cancer cells. *Chin Med J (Engl)* **124**, 1327-1332, (2011).

32 Hayes, J. D. & McMahon, M. Molecular basis for the contribution of the antioxidant responsive element to cancer chemoprevention. *Cancer Lett* **174**, 103-113, (2001).

33 Pobbati, A. V. & Hong, W. Emerging roles of TEAD transcription factors and its coactivators in cancers. *Cancer Biol Ther* **14**, 390-398, (2013).

34 Potapenko, I. O. *et al.* Glycan gene expression signatures in normal and malignant breast tissue; possible role in diagnosis and progression. *Mol Oncol* **4**, 98-118, (2010).

35 Zou, Q. *et al.* Clinicopathological features and CCT2 and PDIA2 expression in gallbladder squamous/adenosquamous carcinoma and gallbladder adenocarcinoma. *World J Surg Oncol* **11**, 143, (2013).

36 Davidson, B. *et al.* Gene expression signatures differentiate uterine endometrial stromal sarcoma from leiomyosarcoma. *Gynecol Oncol* **128**, 349-355, (2013).

37 Azeez, J. M. *et al.* Progesterone regulates the proliferation of breast cancer cells - in vitro evidence. *Drug Des Devel Ther* **9**, 5987-5999, (2015).

38 Alinezhad, S. *et al.* Validation of Novel Biomarkers for Prostate Cancer Progression by the Combination of Bioinformatics, Clinical and Functional Studies. *PLoS One* **11**, e0155901, (2016).

39 Wang, Y. Transcriptional Regulatory Network Analysis for Gastric Cancer Based on mRNA Microarray. *Pathol Oncol Res*, (2017).

40 Xu, G. *et al.* Long noncoding RNA expression profiles of lung adenocarcinoma ascertained by microarray analysis. *PLoS One* **9**, e104044, (2014).

41 Heerema-McKenney, A. *et al.* Clinical, immunophenotypic, and genetic characterization of small lymphocyte-like plasma cell myeloma: a potential mimic of mature B-cell lymphoma. *Am J Clin Pathol* **133**, 265-270, (2010).

42 Stahl, P. R. *et al.* THSD7A Expression in Human Cancer. *Genes Chromosomes Cancer*, (2016).

43 Yoo, E. H. *et al.* Genetic Characteristics of Polycythemia Vera and Essential Thrombocythemia in Korean Patients. *J Clin Lab Anal* **30**, 1061-1070, (2016).

44 Tagi, T. *et al.* Dermokine as a novel biomarker for early-stage colorectal cancer. *J Gastroenterol* **45**, 1201-1211, (2010).

45 Vega, A. *et al.* Evaluating new candidate SNPs as low penetrance risk factors in sporadic breast cancer: a two-stage Spanish case-control study. *Gynecol Oncol* **112**, 210-214, (2009).

46 Yang, T., Li, X. N., Li, X. G., Li, M. & Gao, P. Z. DNAJC6 promotes hepatocellular carcinoma progression through induction of epithelial-mesenchymal transition. *Biochem Biophys Res Commun* **455**, 298-304, (2014).

47 Leroy, C. *et al.* Quantitative phosphoproteomics reveals a cluster of tyrosine kinases that mediates SRC invasive activity in advanced colon carcinoma cells. *Cancer Res* **69**, 2279-2286, (2009).

48 Li, C. P. *et al.* CLDN14 is epigenetically silenced by EZH2-mediated H3K27ME3 and is a novel prognostic biomarker in hepatocellular carcinoma. *Carcinogenesis* **37**, 557-566, (2016).

49 Beadling, C. *et al.* Gene expression of the IGF pathway family distinguishes subsets of gastrointestinal stromal tumors wild type for KIT and PDGFRA. *Cancer Med* **2**, 21-31, (2013).

50 Ghalei, H. *et al.* Hrr25/CK1delta-directed release of Ltv1 from pre-40S ribosomes is necessary for ribosome assembly and cell growth. *J Cell Biol* **208**, 745-759, (2015).

51 He, P. *et al.* Knock-Down of Endogenous Bornavirus-Like Nucleoprotein 1 Inhibits Cell Growth and Induces Apoptosis in Human Oligodendroglia Cells. *Int J Mol Sci* **17**, 435, (2016).

52 Zhang, P. *et al.* Methylation profiling of serum DNA from hepatocellular carcinoma patients using an Infinium Human Methylation 450 BeadChip. *Hepatol Int* **7**, 893-900, (2013).

53 Miao, L. *et al.* SPOCK1 is a novel transforming growth factor-beta target gene that regulates lung cancer cell epithelial-mesenchymal transition. *Biochem Biophys Res Commun* **440**, 792-797, (2013).

54 Qiu, J., Shao, S., Yang, G., Shen, Z. & Zhang, Y. Association of Toll like receptor 9 expression with lymph node metastasis in human breast cancer. *Neoplasma* **58**, 251-255, (2011).

55 Qiu, H. *et al.* EMX2 is downregulated in endometrial cancer and correlated with tumor progression. *Int J Gynecol Pathol* **32**, 193-198, (2013).

56 Okamoto, J. *et al.* EMX2 is epigenetically silenced and suppresses growth in human lung cancer. *Oncogene* **29**, 5969-5975, (2010).

57 Zhang, L. *et al.* MNX1 Is Oncogenically Upregulated in African-American Prostate Cancer. *Cancer Res* **76**, 6290-6298, (2016).

58 Worzfeld, T. & Offermanns, S. Semaphorins and plexins as therapeutic targets. *Nat Rev Drug Discov* **13**, 603-621, (2014).

59 Karayan-Tapon, L. *et al.* Semaphorin, neuropilin and VEGF expression in glial tumours: SEMA3G, a prognostic marker? *Br J Cancer* **99**, 1153-1160, (2008).

60 Xiong, S., Wang, Q., Zheng, L., Gao, F. & Li, J. Identification of candidate molecular markers of nasopharyngeal carcinoma by tissue microarray and in situ hybridization. *Med Oncol* **28 Suppl 1**, S341-348, (2011).

61 Quail, D. F. *et al.* Embryonic protein nodal promotes breast cancer vascularization. *Cancer Res* **72**, 3851-3863, (2012).

62 Weiguang, Y. *et al.* Association of OX40L polymorphisms with sporadic breast cancer in northeast Chinese Han population. *PLoS One* **7**, e41277, (2012).

63 Guan, G. G. *et al.* UBE2D3 is a positive prognostic factor and is negatively correlated with hTERT expression in esophageal cancer. *Oncol Lett* **9**, 1567-1574, (2015).

64 Xu, H. *et al.* Gene expression profiling analysis of lung adenocarcinoma. *Braz J Med Biol Res* **49**, (2016).

65 Wong, Q. W. *et al.* RPL39L is an example of a recently evolved ribosomal protein paralog that shows highly specific tissue expression patterns and is upregulated in ESCs and HCC tumors. *RNA Biol* **11**, 33-41, (2014).

66 Clancy, T. *et al.* Immunological network signatures of cancer progression and survival. *BMC Med Genomics* **4**, 28, (2011).

67 Wilson, T. J., Nannuru, K. C., Futakuchi, M. & Singh, R. K. Cathepsin G-mediated enhanced TGF-beta signaling promotes angiogenesis via upregulation of VEGF and MCP-1. *Cancer Lett* **288**, 162-169, (2010).

68 Anami, K. *et al.* Search for transmembrane protein in gastric cancer by the Escherichia coli ampicillin secretion trap: expression of DSC2 in gastric cancer with intestinal phenotype. *J Pathol* **221**, 275-284, (2010).

69 Stephens, P. J. *et al.* The landscape of cancer genes and mutational processes in breast cancer. *Nature* **486**, 400-404, (2012).

70 Cheng, L. *et al.* FUT family mediates the multidrug resistance of human hepatocellular carcinoma via the PI3K/Akt signaling pathway. *Cell Death Dis* **4**, e923, (2013).

71 Shinmura, K. *et al.* Abnormal Expressions of DNA Glycosylase Genes NEIL1, NEIL2, and NEIL3 Are Associated with Somatic Mutation Loads in Human Cancer. *Oxid Med Cell Longev* **2016**, 1546392, (2016).

72 Chen, Y. *et al.* A NEIL1 single nucleotide polymorphism (rs4462560) predicts the risk of radiation-induced toxicities in esophageal cancer patients treated with definitive radiotherapy. *Cancer* **119**, 4205-4211, (2013).

73 Nicolas, E., Golemis, E. A. & Arora, S. POLD1: Central mediator of DNA replication and repair, and implication in cancer and other pathologies. *Gene* **590**, 128-141, (2016).

74 Khamis, Z. I., Zorio, D. A., Chung, L. W. & Sang, Q. X. The Anti-inflammatory Role of Endometase/Matrilysin-2 in Human Prostate Cancer Cells. *J Cancer* **4**, 296-303, (2013).

75 Tang, W., Dou, T., Zhong, M. & Wu, Z. Dysregulation of Claudin family genes in colorectal cancer in a Chinese population. *Biofactors* **37**, 65-73, (2011).

76 Sun, L. *et al.* Notch Signaling Activation in Cervical Cancer Cells Induces Cell Growth Arrest with the Involvement of the Nuclear Receptor NR4A2. *J Cancer* **7**, 1388-1395, (2016).

77 Jiang, Y. *et al.* Association of FGFR3 and FGFR4 gene polymorphisms with breast cancer in Chinese women of Heilongjiang province. *Oncotarget* **6**, 34023-34029, (2015).

78 Yuan, L. *et al.* Recurrent FGFR3-TACC3 fusion gene in nasopharyngeal carcinoma. *Cancer Biol Ther* **15**, 1613-1621, (2014).
